# Synthetic Image Generation Using the Finite Element Method and Blender Graphics Program for Modeling of Vision-Based Measurement Systems

**DOI:** 10.3390/s21186046

**Published:** 2021-09-09

**Authors:** Paweł Zdziebko, Krzysztof Holak

**Affiliations:** Department of Robotics and Mechatronics, AGH University of Science and Technology, Al. A. Mickiewicza 30, 30-059 Krakow, Poland; zdziebko@agh.edu.pl

**Keywords:** image-based measurement, vision sensor modeling, vision system simulation, image-based reconstruction, finite element method, physics-based computer graphics

## Abstract

Computer vision is a frequently used approach in static and dynamic measurements of various mechanical structures. Sometimes, however, conducting a large number of experiments is time-consuming and may require significant financial and human resources. On the contrary, the authors propose a simulation approach for performing experiments to synthetically generate vision data. Synthetic images of mechanical structures subjected to loads are generated in the following way. The finite element method is adopted to compute deformations of the studied structure, and next, the Blender graphics program is used to render images presenting that structure. As a result of the proposed approach, it is possible to obtain synthetic images that reliably reflect static and dynamic experiments. This paper presents the results of the application of the proposed approach in the analysis of a complex-shaped structure for which experimental validation was carried out. In addition, the second example of the process of 3D reconstruction of the examined structure (in a multicamera system) is provided. The results for the structure with damage (cantilever beam) are also presented. The obtained results allow concluding that the proposed approach reliably imitates the images captured during real experiments. In addition, the method can become a tool supporting the vision system configuration process before conducting final experimental research.

## 1. Introduction

In the recent decade, image processing and computer vision techniques have gained recognition in the engineering society as an important element of inspection and monitoring systems for mechanical and civil engineering structures [1,2,3]. For object surface state assessment, images capture the same information as that usually found by human inspectors. Additionally, image and video files encode full-field displacements or deformation courses. A series of images captured from distinct viewpoints provide information on the 3D structure of the object [4]. Videos, as a time series of images, additionally contain temporal information that can be utilized to find changes in the observed object or obtain dynamic response data if one applies a measurement camera with a frame rate that is sufficiently high [5]. A lot of research reported in the literature has been carried out for image-based remote inspection of civil engineering structures, using high-resolution camera systems mounted on a tripod [3] or unmanned aerial vehicles [6] (UAVs) to record necessary image data. Additionally, one can observe a significant development in artificial intelligence (AI) approaches in computer vision systems [7].

There are two major groups of computer vision applications for structure state assessment: image-based inspection of the surface and vision-based monitoring to obtain the current static state and/or dynamic behavior of the structure. Researchers have developed methods for the detection of various types of damage: concrete cracks, delamination and spalling of concrete, asphalt cracks, steel fatigue cracks and corrosion [8]. In a recent study [9], the authors proposed a tracking measurement of the full-field surface deformation of large-field recycled concrete-filled steel tube columns via a mark-free, four-ocular, stereoscopic visual system. Achieved results proved the high accuracy of this method.

Earlier developed methods of surface damage detection are heuristic and were designed for specific tasks. However, in these methods, all parameters must be chosen manually based on the image data and the user’s practical knowledge. Deep learning introduction expanded the capability and robustness compared to the classical vision [10]. A lot of studies have been conducted to increase the automation level of image-based damage detection. An interesting application is the structural element recognition algorithm. First, the structural elements are localized in the image (e.g., columns, beams) [11]. Next, damage detection is carried out on each of them, and its severity is evaluated with the application of specific technical standards. The entire inspection process is performed with the application of UAV robots [12]. Object detection methods are also used in other vision system applications, e.g., fruit detection systems, such as that presented by Li et al. [13].

Another big area of image processing application in structure state evaluation is static [14,15] and dynamic vision-based measurement [16,17]. Structural deformation is often computed by means of the digital image correlation (DIC) method, a well-known approach in the field of laboratory testing of mechanical properties [18]. In this approach, a high-contrast visual optical noise pattern has to be placed on the object’s surface before an experiment is carried out to increase the performance of the method. The DIC method has also been applied in the displacement measurement of large-scale civil engineering structures. In such cases, a camera equipped with a telephoto lens is used. Usually, it observes a single point, a marker or a natural feature; however, a larger field of view may be observed also by means of the application of a synchronized camera network [19,20]. Stereovision systems are applied to recover the 3D structure of observed objects [21]. Such systems may also be augmented by RGB-D sensors [22]. Multicamera reconstruction can also be performed using even more cameras. For example, Chen et al. [23] and Tang et al. [24] used four-camera vision systems for a tube-like shape object reconstruction.

Most often, the development of image processing algorithms requires access to a large amount of test data. These are video sequences or single images showing objects whose displacement or change in shape is the subject of detection. The structure is usually equipped with visual markers or has an applied optical noise on its surface. This requirement is related to how the algorithms determine changes in the shape of the structure in the image. This is performed by measuring the position of individual markers on the system under study. The design and development of damage detection require vision data (such as images or video sequences) of analyzed structure. Usually, it is necessary to collect data for loaded and unloaded states, as well as for various damage scenarios. This approach requires careful preparation of laboratory setups and performing time-consuming experiments, as damaged structures are not common in practice. It requires the preparation of the test stand, i.e., sample manufacturing, configuration of the test rig and the vision system. Dynamic experiment observations recorded with the use of high-speed cameras are particularly time-consuming due to the large amount of data recorded.

A lot of progress has been made in the field of computer graphics and augmented reality that has allowed the generation of photorealistic images and videos. In the literature, such data have been used to train deep neural networks for the segmentation of images and object classification problems [25]. This significantly increased the available sizes of training data sets for neural networks and allowed the introduction of more variable imaging conditions for synthetic cameras. Two approaches to the generation of synthetic images are available. The first approach uses game engines to render images in a shorter time but with limited capabilities for realism, and the second approach allows rendering scenes using a physics-based ray tracing algorithm to produce high-quality, photorealistic images. However, this approach requires more computational cost. Synthetic images generated in one of these approaches provide a structural model with controllable and repeatable loading and damage conditions. Additionally, the effects of external lighting and vision system optical parameters can be easily simulated. A practical implementation of this idea was presented in the work by Spencer et al. [1]. The authors presented a method of using physical-based models of structures and synthetic image generation to obtain images of structures with different damage conditions. They used synthetic data of a miter gate and a deep neural network to identify changes occurring on the gate. The generated training set of images included models of various damage conditions such as cracks and corrosion. Synthetic images in the data set were generated under different environmental factors such as variable lighting conditions and vegetation growth. 

The results of the review presented in [25] indicate that the currently used methods of synthetic image generation are most often based on existing graphics engines dedicated to games or embedded algorithms in rendering programs, e.g., in Blender or Autodesk Maya. It should be emphasized that the main goals of these solutions are visual effects. When it is more important to reflect the actual deformation of the structure (e.g., under loads), the available engines may not be sufficient and reliable solutions. To the best of our knowledge, there are no published validation results that can definitely prove that the mechanical deformations in synthetic images are realistic. 

This article addresses this challenge. As part of this work, the authors propose a solution that aims at synthetic image generation based on finite element analysis results, which exemplifies the novelty of this work. In general, the proposed solution consists of using a model formulated with the finite element method (FEM), which is a widely recognized simulation method that precisely simulates the deformation of the studied model. Then, the graphics program Blender is used to generate synthetic images using computer resources (renderings). 

The limitations of the proposed method are mainly related to the detailed representation of the simulated scene. The complicated shape of the observed structure requires the formulation of a dense FEM mesh. This results in a significant extension of computation time. Moreover, another limitation of the applied method is the complex state of illumination of the scene and a detailed representation of texture reflectance, as well as the size of the generated image. All these factors increase the rendering time of the scene. Overlapping these factors can make synthetically producing images more time-consuming than conducting real experiments in extreme cases. However, in most practical applications, these limitations are limited, and the computational efficiency of the followed numerical approach is satisfactorily high.

FEM simulations are widely used in the analysis of engineering problems, including multidomain simulations, in which an important role is played by coupling between various physical domains, such as thermal and mechanical [26]. This method is also used to analyze other problems, such as metal forming [27] or the analysis of components made with composite materials [28]. The joint simulation of the Blender graphics program and the FEM has been presented recently [29]. Nevertheless, the Blender program was only used to define a finite element mesh. The second example of Blender integration, this time in the computer-aided design (CAD) environment, was presented by Vasilev et al. [30]. The images were rendered in Blender, but the whole process did not involve FEM calculations to obtain the deformation of objects. 

The novelty of the simulation approach introduced in this paper is the combination of the FEM method and the ability to render synthetic images in the Blender program. Owing to the use of FEM simulation models, high accuracy of simulated displacements is ensured. Moreover, it is possible to simulate any complex mechanical structure. The authors of this work have developed their own numerical environment. It allows customizing the simulation and automatically renders images of statically or dynamically loaded mechanical structures. In the proposed solution, FEM models are computed in the MSC.Marc solver, which is dedicated to nonlinear analyses (e.g., with contacts) and multidomain couplings. Moreover, owing to the developed simulation setup, the FEM analysis can be individually customized to a specific problem (e.g., modeling of vision markers). The proposed algorithm is discussed in detail in Section 2. Subsequently, Section 3 presents two examples of applications for generating synthetic images. The first example includes experimental validation of the proposed simulation approach, while the second example presents the simulation results for a multicamera system. Section 4 summarizes the presented work.

## 2. Materials and Methods

**Figure 1:**
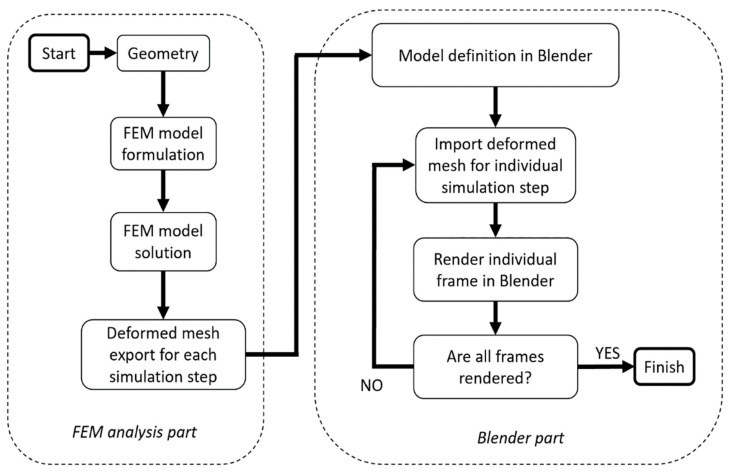
Scheme of the proposed simulation algorithm.

The proposed computing environment uses FEM simulation tools and the Blender graphics program. The diagram of the proposed algorithm is shown in Figure 1. Synthetic images of the structure under study are obtained as a result of the algorithm’s operation. The aim of the proposed method is to synthetically produce images of mechanical structures subjected to loads. It is also necessary to ensure real deformations and parameters of realistic vision systems (lens, camera resolution, lighting conditions). Further use of the images produced can vary. It can be focused on line deflection calculation or defect detection based on image processing. The simulation methodology begins with a definition of a model geometry using a CAD program or directly in the FEM preprocessor. More complex components are usually more convenient to be modeled in a dedicated CAD program.

Next, the FEM model is formulated in the FEM preprocessor. The authors used the Altair HyperMesh program. Essential steps in this stage are as follows: defining a finite element mesh, assigning material parameters and defining the boundary conditions and the load step. The finite elements should be of a solid type (3D elements, e.g., in hexahedral, tetrahedral or pentahedral shape). This requirement is dictated by the requirement to render a closed volume in the next stage of the algorithm’s work. Moreover, to enable the export of the FEM mesh into the Blender graphics program, it is necessary to cover solid elements with a membrane of 2D elements in the model. Its thickness and stiffness should be selected so as not to affect the simulation results. The calculations assumed a membrane thickness of 0.001 mm and a Young’s modulus 10 times lower than the native material of 3D elements. The membrane mesh is deformed together with the native material. It has common nodes with the 3D mesh. Owing to this, and ensuring the unambiguity of the displacements, it is possible to correctly project the deformed mesh in Blender (at a later stage of the algorithm). Degrees of freedom of the elements should correspond to the type of analysis being carried out, e.g., displacements and rotations in nodes for the mechanical domain. In this analysis domain, with the elastic deformation regime, the most important mechanical properties of the material to be modeled are Young’s modulus, Poisson’s ratio and density. The boundary conditions should reflect the actual loads as closely as possible. In most cases for mechanical domain analysis, there are pressure loads and constraints on appropriate degrees of freedom. The equation of the formulated FEM model in the general form is given by Equation (1).

(1) Ku=F

where:

K—global stiffness matrix;

u—global displacement vector;

F—global vector of nodal forces.

The definition load step describes the sequence in which the boundary conditions are applied to the structure. It also defines the type of analysis to be performed (e.g., static, quasi-static or dynamic transient). Then, the formulated FEM model is solved using a dedicated solver. In this step, the global displacement vector is determined. The solution proposed by the authors of this work is based on the MSC.Marc solver. The convergence condition of the analysis dictates the step size in the FEM simulation. The second criterion for the minimum number of steps in the FEM load step is imposed by the number of required rendered frames (in Blender). In quasi-static problems, it is usually two frames (before and after load application). 

The next step of the algorithm is to export the deformed finite element mesh to a *.STL file for individual time steps in the simulation. The authors developed the scripts to automate exporting the current state of the structure’s deformation in the required time intervals, which is especially important for simulating a dynamic phenomenon. The mesh of the system under study must be divided into several parts. The number of exported meshes depends on the number of textures used further in the algorithm in Blender. In other words, if the resulting rendered image presents, for example, three textures or colors on the object, then the export of the deformed mesh must be done separately for each of these areas. 

Next, the Blender program is involved in the proposed solution. The essential Blender model components and parameters are defined at first. These are: cameras (position, orientation, sensor size, lens focal length), lighting points (light shade, intensity) and textures that are later applied to the imported mesh. These parameters should correspond to real-file experimental conditions. In the next step, the import process of the deformed mesh begins. The rendering of a single frame/image is performed next and is repeated for all required frames. The task becomes much more time-consuming when it is necessary to generate a large number of images. This can be necessary for synthetic images generation of dynamic problems. A Python script was developed to automate this task.

As discussed before, the purpose of the proposed method is to produce synthetic images. Nevertheless, it seems necessary to report on potential application areas of the method. Therefore, in Section 3, three case studies of the use of the FEM+Blender simulation are presented. These are not the only possible fields of application of the proposed approach but only representative examples. They are limited to relatively simple mechanical structures but take into account various load conditions and the occurrence of damage. The application of this approach for arbitrarily complex objects is possible but requires the formulation of complex FEM and Blender models. In the presented case studies, the proposed FEM+Blender simulation is limited to quasi-static cases.

## 3. Results and Discussion

This section presents three case studies of the use of the proposed numerical simulation approach. The first example shows the tower crane structure. In this case study, the experimental validation of the deflection value is performed for a simple load case applied to the sample. The second example is based on the same tower-crane-like structure sample but loaded by forces acting together in two directions. This example shows the possibility of simulating a multicamera system, and the displacement measurement is performed in 3D. The third example concerns a relatively simple structure, i.e., cantilever beam, but it demonstrates the use of the simulation approach to generate images of structures with introduced damage (modeled as material discontinuities).

### 3.1. Case Study: Tower Crane Structure

**Figure 2:**
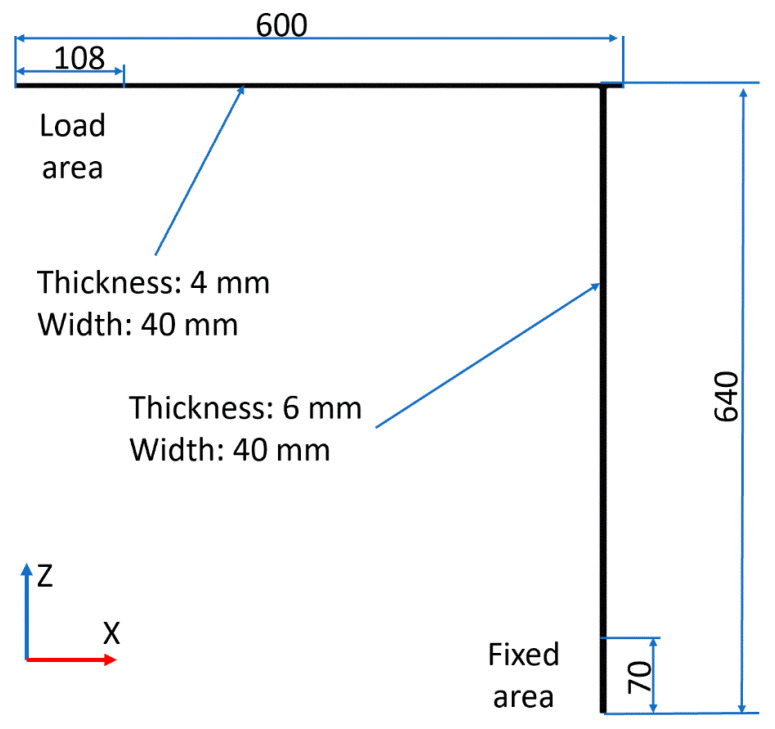
Scheme of the examined tower-crane-like structure.

This subsection presents an example of the utilization of the proposed FEM+Blender simulation for synthetic image generation. In parallel, the experiments were performed to validate the numerical approach. The shape of the used sample (same in the numerical model and experiments) corresponds to the tower-crane-like structure, which consists of two steel beams welded together. The adopted dimensions of the sample are shown in Figure 2. The cross-section of the vertical beam (6 × 40 mm) is greater than the horizontal section (4 × 40 mm) to make the analyzed structure more complex, as it has nonuniform stiffness. The boundary conditions applied to the structure are as follows: clamp constraints on the 70 mm section at the base of the tower crane, and the load constraints in the Z-direction on the 108 mm section from the free end of the sample.

**Figure 3:**
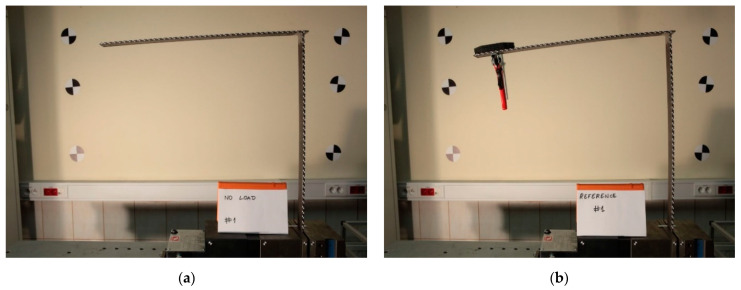
Images of the structure captured in the tests: unloaded state (**a**) and loaded state (**b**).

During the experiments, a specimen was fixed in a pneumatic clamp. Standard vision markers in the shape of black and white boxes were used. They were placed on the side surface of the sample. The vision markers are used to determine the deflection of the structure at a later stage in this work using image processing algorithms. Their shape and placement were the same as in the numerical model discussed in more detail in Section 3.1.1. In the case of the experiment, the vision markers were also glued to stationary objects, such as walls, in order to check whether the camera tripod was moved during the tests. Flat weights mounted near the free end of the sample loaded the structure. The images were captured using one full-frame 21.1 MP Canon EOS 5D Mark II digital single-lens reflex camera (DSLR) with a focal length that amounted to the value of 30 mm. One camera is sufficient to measure the deflection in one plane in the case of an elementary load case. The camera’s position was arbitrarily chosen to allow observing the whole structure and was fixed during experiments. The camera was located in such a way that the lens’ axis was perpendicular to the plane appointed by the tower crane structure and was placed 1 m away from the sample. The focal length was chosen to allow registering the whole sample with its nearest surroundings. A two-point halogen lamp positioned on the left side behind the camera, slightly below the lens axis, illuminated the scene. The vision system and the sample were not disassembled during the experiments. Therefore, the position of the sample, illuminators, camera and tripod remained the same during the experiment. The experiments were performed without access to natural light that could have altered the illumination level of the scene. Example captured photos of an unloaded and a loaded sample are shown in Figure 3a,b, respectively. In order to compute the deflection of the crane structure, pixel coordinates of the markers’ centers were detected in the reference image (unloaded state) and images of the structure after load application. Each marker’s center was detected using the Harris corner detector, and its consecutive displacement under the load was tracked by means of a digital image correlation (DIC) algorithm. The scale coefficient (mm/pix) was computed using the known distance between chosen markers on the horizontal part of the crane. Its value was equal to 0.19 mm/pix. 

#### 3.1.1. Numerical Model Validation

**Figure 4:**
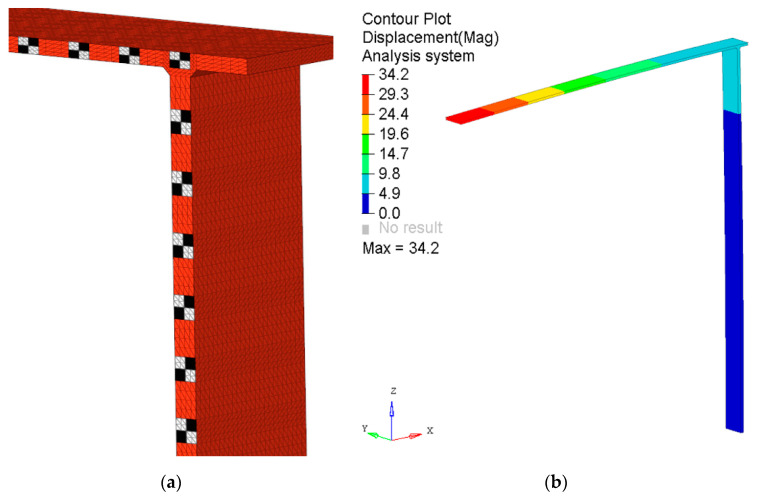
FEM model: close-up view of a finite element mesh in the area of connection of a vertical and horizontal beam (**a**) and the results of deflection of the tested structure (**b**).

According to the presented simulation algorithm’s description (Section 2), the development of the simulation model began with the CAD model definition. The modeled geometry was consistent with the experimental sample, and the diagram is shown in Figure 2. In the next stage of the process, the FEM model was formulated. Figure 4a presents a view of a finite element mesh in the area of the connection of two steel beams. It should be emphasized that the color of the elements used in the visualization is not essential. As mentioned earlier, it is necessary to group finite elements due to the texture applied to them later in the Blender program. In the presented case, this requirement was achieved by renumbering the finite elements so that the relevant fragments of the examined structure had ID numbers from known ranges. Owing to that, it was possible to export selected parts of the mesh, on which different textures (white, black, metallic) were then applied in the Blender graphics program.

The FEM model also defined material parameters and boundary conditions that corresponded to the experiment. The conducted analysis was quasi-static. Therefore, the results of structure deformation were determined for two calculation steps: before and after the load application. The displacement results are shown in Figure 4b. 

**Figure 5:**
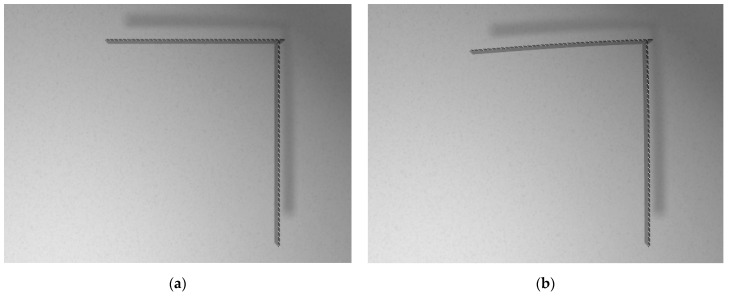
Renders generated using the proposed algorithm: unloaded structure (**a**) and loaded structure (**b**).

According to the simulation algorithm, the exported FEM mesh was then imported into the model in Blender. White and black textures were applied to the appropriate fragments of the structure to model the proper places of the video markers. For the remaining areas of the tower crane, a texture imitating the steel surface was adopted with a gray color and increased reflectivity. Rendering was performed using an Nvidia GeForce GTX 1060 6 GB graphics processing unit (GPU) and lasted 170 s for a single image. Synthetically produced nondeformed and deformed structure renders are shown in Figure 5a,b, respectively. 

The modeled camera had a sensor of 36 × 24 mm, and the utilized lens was set to a focal length of 30 mm to keep the simulation consistent with the experimental setup. The rendered image size was limited to 5616 × 3744 pix. Similar to the experimental case, the synthetic images were processed in order to detect positions of markers in the reference image and images generated after load application on the structure. Initial positions of markers (both in numerical and experimental cases) were found by the Harris corner detector and tracked by means of the DIC method. The computed scale coefficient was equal to 0,21 mm/pix. The value was different than in the experimental case. The main reason for this discrepancy is that the position and orientation of the camera were not exactly the same as in the real laboratory setup. 

**Figure 6:**
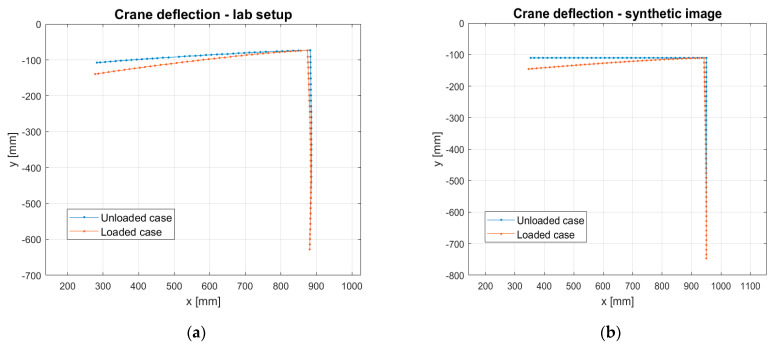
Results of the comparison of the tower crane deflection lines for the experimental data (**a**) and for the FEM + Blender (**b**) simulation (renders).

After computation was carried out, vision marker positions were obtained for the experimental data (real images) and for synthetically simulated data (FEM + Blender approach). Results of the crane’s deflection are presented in Figure 6. The value of the maximum deflection for the experimental case was 32.68 mm, and that measured using synthetic images amounted to a value of 32.76 mm. The relative difference between results was 0.26%.

It can be observed that the structure is deformed slightly differently in the absence of load when comparing experimental and simulation results. This is due to the fact that the FEM model ignored gravity and the fact that during the manufacturing of experimental samples, there was material shrinkage in the welding process and no exact 90° angle between the beams. It should also be emphasized that the conducted experiments and simulations were carried out in the elastic (linear) regime of deformations. Nevertheless, the most important parameter in this case study is the deformation after the load is applied, and for this parameter, the simulation compliance with the experiment is very high (0.26% error). Therefore, it can be concluded that a correctly adjusted method for synthetic images creation allows obtaining realistic pictures of the analyzed structure. The method can therefore be used to generate vision data without performing experiments.

### 3.2. Case Study: Tower Crane Structure with Complex Load Case

**Figure 7:**
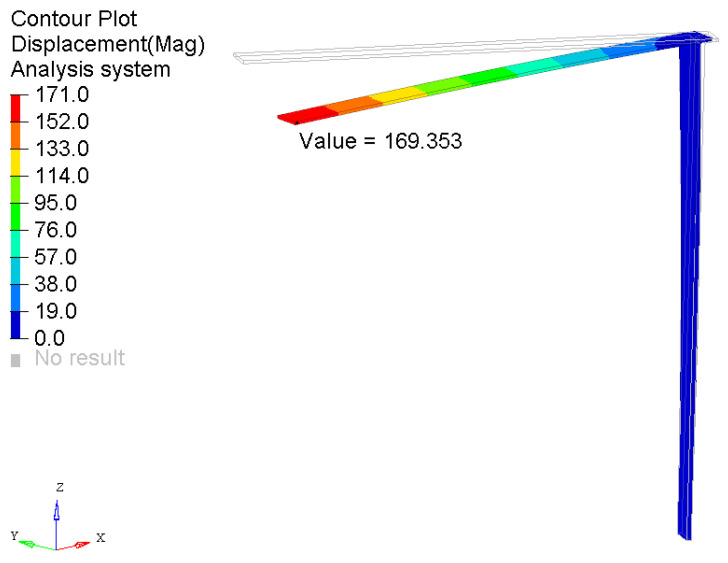
Results of the displacement of the tower crane structure subjected to complex load.

This example is presented to demonstrate the possibility of simulating the multicamera system, which is necessary to be used to measure the deflection of a tower crane structure with an applied complex load case. The resulting deflection, which occurs in the 3D space, cannot be measured by a single camera system in this case. The shape of the sample is the same as that introduced in Section 3.1, but the load acts in two directions: 192 N on the *Y*-axis and 13 N on the *Z*-axis, according to Figure 2. Load is applied to the free end of the sample. Moreover, the sample is rotated 30° on the *Z*-axis. This is performed to make the analysis more complex and realistic. In this case study, only the numerical approach was considered, as described in Section 3.1. The method was successfully validated. The resulting magnitude of displacement of the free end based on the FEM results equals 169.353 mm, as depicted in Figure 7. The original shape of the structure in the reference state is also depicted by thin lines. 

**Figure 8:**
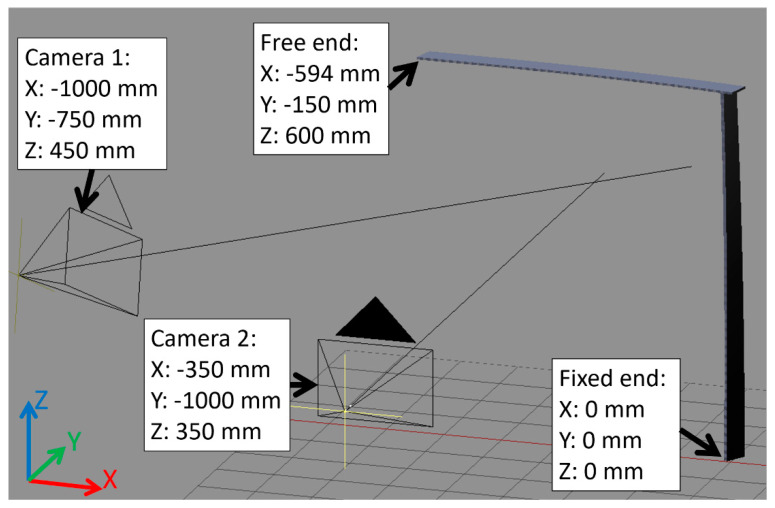
Spatial arrangement of cameras and the tower crane sample in multicamera simulation.

The FEM analysis results were then used to produce renders according to the procedure introduced in Section 2. The multicamera system was composed of two cameras of the same type as used in Section 3.1. The utilized lens was set to a focal length of 30 mm for Camera 1 and 32 mm for Camera 2. The spatial arrangement of the cameras and simulated sample are shown in Figure 8. Both cameras were directed toward the observed sample. 

**Figure 9:**
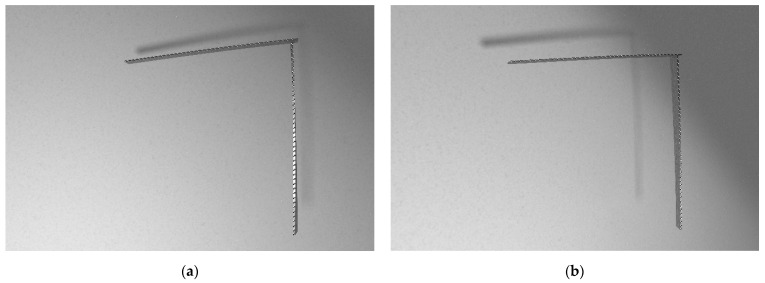
Renders generated using the proposed algorithm of the loaded sample: rendered by Camera 1 (**a**) and rendered as captured by Camera 2 (**b**).

As a result of the rendering procedure, the images obtained from the two virtual cameras were generated for the reference (unloaded) and loaded states. The resulting rendered images are presented in Figure 9a,b (loaded sample captured by Camera 1 and loaded sample captured by Camera 2, respectively). Simultaneous deflection of the horizontal part and the twist of the vertical part can be easily observed.

The purpose of using two cameras is to carry out the 3D reconstruction of the tested specimen. To be able to carry out this process, it is necessary to compute both intrinsic and extrinsic camera matrices that describe the projection equation and the position and orientation relationship between the two cameras, respectively. Pinhole camera models were used to model both cameras in the system (Equation (2)).

(2) $$\lambda_{1}x_{1}=K_{1}P_{0}X\;\lambda_{2}x_{2}=K_{2}P_{0}GX$$

where, assuming that the 3D reconstruction will be carried out in the coordinate frame of Camera 1: **K_1_**, **K_2_**—intrinsic camera matrices of Cameras 1 and 2; **G**–extrinsic matrix of the camera system; **X**—3D coordinates of scene points; **x_1_**, **x_2_**—coordinates of image points in the image planes of the two cameras corresponding to points **X**; *λ*_1_, *λ*_2_—corresponding scales; **P_0_**—standard projection matrix. 

The calibration was carried out based on images of the standard calibration planar target in the form of a chessboard table captured by two cameras simultaneously. In the case of synthetic images, a calibration table model of 8 × 9 fields with dimensions of 40 × 40 mm was used. The computed set of renders, consisting of 21 calibration board images for each of the cameras, was used as input data in the stereo camera system calibration algorithm in the Computer Vision Toolbox of the MATLAB programming environment. 

**Figure 10:**
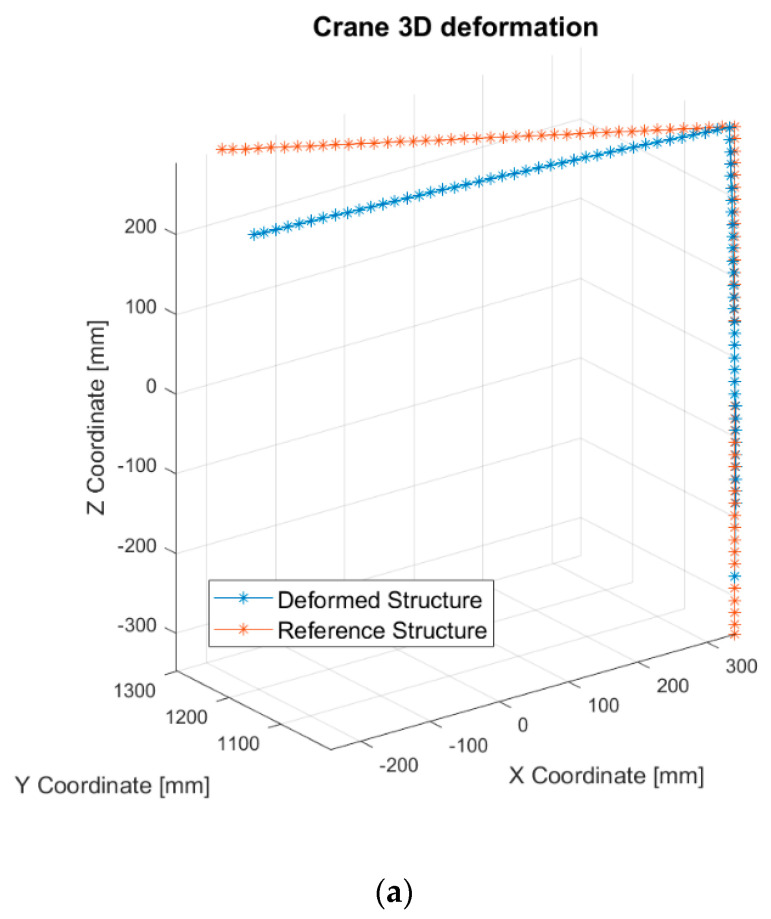

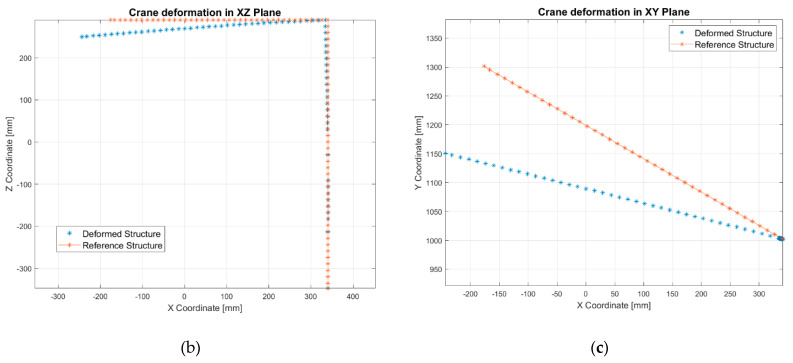
Three-dimensional (3D) reconstruction of the crane structure obtained using image data from the two-camera system: reference case (red) and after application of the load (blue). (**a**) Three-dimensional (3D) reconstruction and two projections; (**b**) projection on the XZ plane; (**c**) projection on the XY plane.

After the calibration, corresponding pairs of markers were detected on two images using the Harris corner detection algorithm. With known internal and external parameters of the two-camera system and a set of corresponding feature points, the metric 3D coordinates of the markers were obtained in the first camera reference frame. Computation was carried out using the direct linear transformation (DLT) algorithm [31] utilizing epipolar constraints. The resulting 3D structure is shown in Figure 10a. Figure 10b,c presents the reconstruction projected on two perpendicular planes to better visualize the 3D deformation of the specimen. The displacement of the free end of the crane structure obtained from the vision method was equal to 169.908 mm. The error with respect to the true displacement (obtained in the FEM method, equal to 169.353 mm) amounted to 0.32%. 

### 3.3. Case Study: Cantilever Beam with Damage

**Figure 11:**
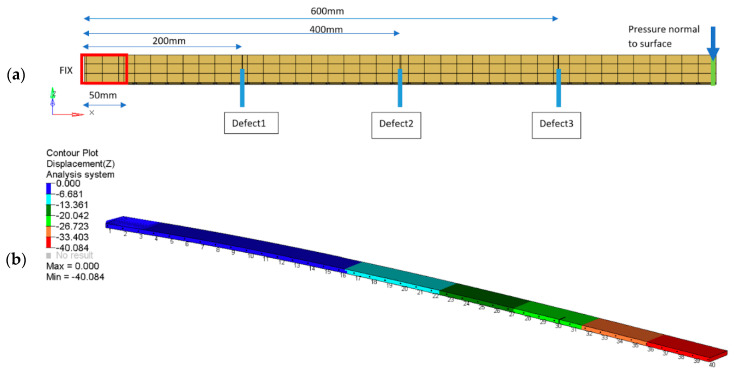
Analyzed beam structure: model scheme (**a**) and results of the beam deflection under load (**b**).

The third analyzed example consists of simulating a beam with damage. The analysis is limited to the simple type of defect, which is a discontinuity of the material (incision introduction). The dimensions of the simulated structure are as follows: 800 mm length, 40 mm wide, 4 mm thickness. Figure 11a shows the location of the modeled defects and the areas of the constraint and load application. The assumed damages were modeled as notches with a 1 mm width and a depth that corresponds to one-third of the beam’s width (13.33 mm). In this case study, only synthetically generated images were considered. The performed analysis was of the quasi-static type. The results were obtained for the unloaded case and after the load was applied. The case without damage was treated as the reference case. Figure 11b shows the deflection results of a beam with three defects obtained by the FEM solution.

**Figure 12:**
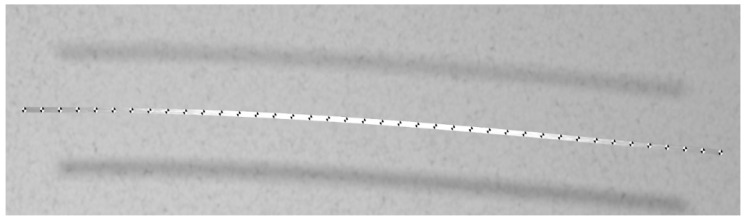
Render obtained for the adopted camera system.

The procedure for generating synthetic images remains the same as before. The virtual camera was positioned centrally in front of the test sample. The modeled camera had a sensor of 36 × 24 mm, which simulated the DSLR Canon 5D Mark II sensor size as in previous case studies. The utilized lens was set to a focal length of 43 mm, and the rendered image size was limited to 2560 × 600 pix. The render shown in Figure 12 was obtained as one of the results of the analysis.

**Figure 13:**
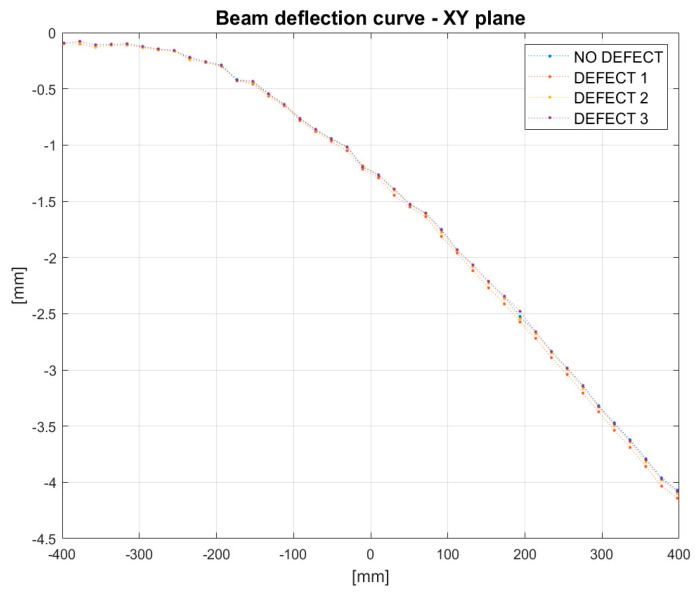
Deflection curve-based deflection on synthetic images for different damage conditions introduced.

Positions of markers on the reference image were detected, as in the previous examples, using the Harris corner detector. The displacement of all markers caused due to damage introduced to the beam’s structure was tracked using the DIC algorithm. As a result, deflection curves of the beam under load were obtained. Figure 13 presents the scaled beam’s deflection for different levels of the introduced damage obtained by image processing. 

**Figure 14:**
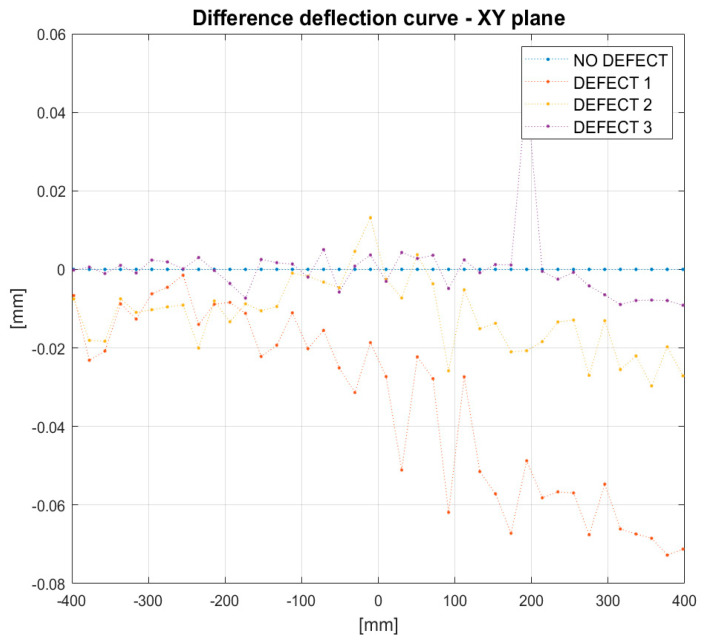
Difference in deflection of the beam under load for different damage conditions.

An impact of damage on the deflection curve can be seen in Figure 14. The curve presents a difference between the deflection of the undamaged beam and the deflection after the introduction of three damage scenarios. The ‘NO DEFECT’ line was introduced as a reference. For all damage cases, the trends of difference deflection curves can be clearly seen in the figure, despite the presence of noise. The ‘DEFECT 1’ line corresponds to the damage introduced at a distance of 200 mm from the clamped side of the beam. In the camera frame, it corresponds to an x-distance equal to −200 mm. One can notice that the red curve changes character near the points x = −200 mm. The horizontal curve becomes inclined. Similar behavior can be seen by the difference in deflection curves corresponding to the other damage cases. ‘DEFECT 2’ and ‘DEFECT 3’ correspond to damage at the distances of 400 mm and 600 mm from the clamped end of the beam, respectively. The curves representing the difference in deflection of the beam change the slope approximately at points x = 0 mm and x = 200 mm, which coincides with the position of damage in the camera coordinate frame. This suggests that the change of the slope can be used as an indicator of damage position.

## 4. Conclusions

This paper presents a new computer simulation methodology for synthetic image creation. The solution is dedicated to the presentation of mechanical structures under the influence of external forces. This approach uses the FEM to determine the deformation of the tested system under the influence of loads acting on it. The resulting deformed finite element mesh is imported into the Blender environment, and synthetic images are rendered using a GPU. Camera and lens parameters, light source, reflections, material textures and shadows are considered during the rendering process. The proposed approach can produce synthetic data that can be used as the input data to test image processing algorithms, and this is the main area of application of this approach. In some cases, the proposed methodology can introduce a significant reduction in the time required to obtain the data compared to the actual experiments. The authors are aware that the application of the proposed approach does not allow for the omission of experiments but may, for example, help in choosing the appropriate camera positioning during the actual experiment and introduce significant time savings.

The aim of the proposed method, the same as for other simulation methods, is to obtain certain results in a numerical manner with the use of computer resources. The obtained video data can be further analyzed, depending on the specific need. The proposed simulation approach can be used to generate synthetic data, e.g., to increase training sets for neural networks to interpret data in images. It may also help in selecting components of the target configuration of the vision measurement system in engineering operation conditions. In such an application, it is necessary to predict the expected displacements of the structure’s component, which is provided by our solution. It is also necessary to adjust vision system settings to make the structure’s displacements observable by the vision measurement system, which can also be simulated by the developed numerical environment. 

The presented application examples of the proposed algorithm at the moment are limited to the simulation of static scenes, such as structural deflection under load. Sample analyses were presented using generated synthetic vision data. The study focused on determining the deflection of the tested structure with the introduced damage (limited to material discontinuity) and simple and complex load conditions. The last case study allowed generating images of the structure deflected in 3D. The obtained images allowed for the 3D reconstruction of the sample in the simulated multicamera system. The selected results in the paper have been successfully validated experimentally. 

As part of the ongoing further work, the authors develop a simulation environment for synthetic video sequence generation for dynamic phenomena. Such data are needed to test, for example, motion magnification algorithms or normal modes identification algorithms based on video sequences. The simulation setup will be also improved to include depth of field effects in renderings. Initial work in this area has been undertaken, but it requires a thorough quantitative analysis, which is a part of further research.

## Data Availability

The data presented in this study are available on request from the corresponding author.
